# Diaqua­(1,4,8,11-tetra­aza­cyclo­tetra­decane-κ^4^ *N* ^1^,*N* ^4^,*N* ^8^,*N* ^11^)copper(II) dihepta­noate dihydrate

**DOI:** 10.1107/S1600536810025687

**Published:** 2010-07-07

**Authors:** Nur Syamimi Ahmad Tajidi, Norbani Abdullah, Zainudin Arifin, Kong Wai Tan, Seik Weng Ng

**Affiliations:** a Department of Chemistry, University of Malaya, 50603 Kuala Lumpur, Malaysia

## Abstract

The Cu^II^ atom in the title salt, [Cu(C_10_H_24_N_4_)(H_2_O)_2_][CH_3_(CH_2_)_5_CO_2_]_2_·2H_2_O, is chelated by the four N atoms of the 1,4,8,11-tetra­aza­cyclo­tetra­decane (cyclam) ligand and is coordinated by two water mol­ecules in a tetra­gonally Jahn–Teller-distorted octa­hedral geometry. The Cu^II^ atom lies on a center of inversion. The cations, anions and uncoordinated water mol­ecules are linked by N—H⋯O and O—H⋯O hydrogen bonds, forming a layer structure parallel to (100). The alkyl chain of the anion is disordered over two positions in a 0.82 (1):0.18 (1) ratio.

## Related literature

For related diaqua­(1,4,8,11-tetra­aza­cyclo­tetra­deca­ne)copper carboxyl­ates, see: Lindoy *et al.* (2003 ▶); Hunter *et al.* (2005 ▶).
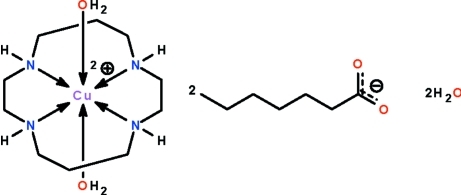


## Experimental

### 

#### Crystal data

[Cu(C_10_H_24_N_4_)(H_2_O)_2_](C_7_H_13_O_2_)_2_·2H_2_O
*M* *_r_* = 594.28
Monoclinic, 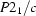

*a* = 11.7257 (6) Å
*b* = 9.9426 (5) Å
*c* = 13.4573 (7) Å
β = 103.1363 (7)°
*V* = 1527.85 (14) Å^3^
*Z* = 2
Mo *K*α radiation
μ = 0.76 mm^−1^
*T* = 100 K
0.35 × 0.25 × 0.15 mm

#### Data collection

Bruker SMART APEX diffractometer
Absorption correction: multi-scan (*SADABS*; Sheldrick, 1996 ▶) *T*_min_ = 0.776, *T*_max_ = 0.894
14358 measured reflections
3506 independent reflections
3140 reflections with *I* > 2σ(*I*)
*R*_int_ = 0.022

#### Refinement

*R*[*F* ^2^ > 2σ(*F* ^2^)] = 0.028
*wR*(*F* ^2^) = 0.079
*S* = 1.06
3506 reflections
212 parameters
12 restraints
H atoms treated by a mixture of independent and constrained refinement
Δρ_max_ = 0.81 e Å^−3^
Δρ_min_ = −0.31 e Å^−3^

### 

Data collection: *APEX2* (Bruker, 2009 ▶); cell refinement: *SAINT* (Bruker, 2009 ▶); data reduction: *SAINT*; program(s) used to solve structure: *SHELXS97* (Sheldrick, 2008 ▶); program(s) used to refine structure: *SHELXL97* (Sheldrick, 2008 ▶); molecular graphics: *X-SEED* (Barbour, 2001 ▶); software used to prepare material for publication: *publCIF* (Westrip, 2010 ▶).

## Supplementary Material

Crystal structure: contains datablocks global, I. DOI: 10.1107/S1600536810025687/bt5285sup1.cif

Structure factors: contains datablocks I. DOI: 10.1107/S1600536810025687/bt5285Isup2.hkl

Additional supplementary materials:  crystallographic information; 3D view; checkCIF report

## Figures and Tables

**Table 1:** Selected bond lengths (Å)

Cu1—N1	2.026 (1)
Cu1—N2	2.025 (1)
Cu1—O1w	2.499 (1)

**Table 2:** Hydrogen-bond geometry (Å, °)

*D*—H⋯*A*	*D*—H	H⋯*A*	*D*⋯*A*	*D*—H⋯*A*
N1—H1⋯O1^i^	0.86 (1)	2.30 (1)	2.983 (2)	137 (2)
N2—H2⋯O2	0.86 (1)	2.12 (1)	2.924 (2)	156 (2)
O1w—H11⋯O2	0.83 (1)	1.93 (1)	2.730 (2)	162 (2)
O1w—H12⋯O2w	0.83 (1)	1.95 (1)	2.777 (2)	174 (2)
O2w—H21⋯O1^ii^	0.83 (1)	2.02 (1)	2.833 (2)	166 (2)
O2w—H22⋯O1^i^	0.84 (1)	1.91 (1)	2.743 (2)	171 (2)

Symmetry codes: (i) 
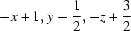
; (ii) 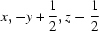.

**Fig. 1.**
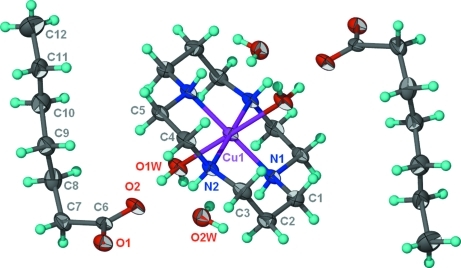
Anisotropic displacement ellipsoid plot (Barbour, 2001) of [Cu(H2O)2(C10H24N4)]2+ 2[CH3(CH2)5CO2]-.2H2O at the 70% probability level; hydrogen atoms are drawn as spheres of arbitrary radius. The disorder in the alkyl chain is not shown.

## supplementary crystallographic information

### Comment

The copper(II) ion forms a number of complexes with 1,4,8,11-tetraazacyclotetradecane in which the metal atom is coordinated by the four amino donor-atoms of the cyclic ligand. Among the carboxylate derivatives, neither the acetate nor the benzoate ions bind directly with the copper atom. The copper atom is coordinated instead by water molecules so that the carboxylate group interacts indirectly with the metal atom through the coordinated water molecules (Hunter *et al.*, 2005; Lindoy *et al.*, 2003). The copper(II) atom in the salt, [Cu(H_2_O)_2_(C_10_H_24_N_4_)]^2+^ 2[CH_3_(CH_2_)_5_CO_2_]^-.^2H_2_O (Scheme I), is chelated by the four nitrogen atoms of the cyclam ligand and is coordinated by two water molecules in a Jahn-Teller type of tetragonally distorted octahedral geometry. The copper atom lies on a center of inversion (Fig. 1). The cations, anions and lattice water molecules are linked by N–H···O and O–H···O hydrogen bonds to form a layer structure.

### Experimental

1,4,8,11-Tetraazacyclotetradecane (0.50 g, 2.50 mmol) dissolved in ethanol (25 ml) was mixed with a suspension of copper heptanoate (0.80 g, 2.5 mmol) in ethanol (50 ml) to give a purple solution. The solution was heated for an hour and then filtered. Prismatic crystals separated from the solution when it was left to cool slowly.

### Refinement

Carbon-bound H-atoms were placed in calculated positions (C—H 0.95 to 0.99 Å) and were included in the refinement in the riding model approximation, with *U*(H) set to 1.2 to 1.5*U*(C).

The amino and water H-atoms were located in a difference Fourier map, and were refined with distance restraints of N–H···O 0.86±0.01, O–H 0.84±0.01 Å; their isotropic displacement parameters were freely refined.

The alkyl chain of the carboxylate ion is disordered over two positions; the disorder refined to an 82 (1):18 (1) ratio. Bond distances for each pair of bonds were restrained to within 0.01Å of each other. The displacement parameters of the primed atoms were constrained to be equal of those of the unprimed ones.

### Crystal data

**Table:**

[Cu(C_10_H_24_N_4_)(H_2_O)_2_](C_7_H_13_O_2_)_2_·2H_2_O	*F*(000) = 646
*M**_r_* = 594.28	*D*_x_ = 1.292 Mg m^−^^3^
Monoclinic, *P*2_1_/*c*	Mo *K*α radiation, λ = 0.71073 Å
Hall symbol: -P 2ybc	Cell parameters from 8003 reflections
*a* = 11.7257 (6) Å	θ = 2.6–28.3°
*b* = 9.9426 (5) Å	µ = 0.76 mm^−^^1^
*c* = 13.4573 (7) Å	*T* = 100 K
β = 103.1363 (7)°	Block, purple
*V* = 1527.85 (14) Å^3^	0.35 × 0.25 × 0.15 mm
*Z* = 2	

### Data collection

**Table:**

Bruker SMART APEX diffractometer	3506 independent reflections
Radiation source: fine-focus sealed tube	3140 reflections with *I* > 2σ(*I*)
graphite	*R*_int_ = 0.022
ω scans	θ_max_ = 27.5°, θ_min_ = 1.8°
Absorption correction: multi-scan (*SADABS*; Sheldrick, 1996)	*h* = −15→15
*T*_min_ = 0.776, *T*_max_ = 0.894	*k* = −12→12
14358 measured reflections	*l* = −17→17

### Refinement

**Table:**

Refinement on *F*^2^	Primary atom site location: structure-invariant direct methods
Least-squares matrix: full	Secondary atom site location: difference Fourier map
*R*[*F*^2^ > 2σ(*F*^2^)] = 0.028	Hydrogen site location: inferred from neighbouring sites
*wR*(*F*^2^) = 0.079	H atoms treated by a mixture of independent and constrained refinement
*S* = 1.06	*w* = 1/[σ^2^(*F*_o_^2^) + (0.0406*P*)^2^ + 0.7328*P*] where *P* = (*F*_o_^2^ + 2*F*_c_^2^)/3
3506 reflections	(Δ/σ)_max_ = 0.001
212 parameters	Δρ_max_ = 0.81 e Å^−^^3^
12 restraints	Δρ_min_ = −0.31 e Å^−^^3^

### Fractional atomic coordinates and isotropic or equivalent isotropic displacement parameters (Å^2^)

**Table:**

	*x*	*y*	*z*	*U*_iso_*/*U*_eq_	Occ. (<1)
Cu1	0.5000	0.5000	0.5000	0.01672 (8)	
O1	0.37230 (9)	0.57029 (10)	0.88828 (7)	0.0254 (2)	
O2	0.35970 (10)	0.48007 (10)	0.73553 (8)	0.0260 (2)	
O1W	0.35364 (10)	0.35174 (11)	0.55509 (8)	0.0262 (2)	
H11	0.3445 (19)	0.378 (2)	0.6109 (10)	0.047 (6)*	
H12	0.3586 (17)	0.2684 (10)	0.5589 (15)	0.035 (5)*	
O2W	0.38768 (10)	0.07550 (11)	0.57173 (8)	0.0272 (2)	
H21	0.372 (2)	0.031 (2)	0.5184 (11)	0.045 (6)*	
H22	0.4606 (9)	0.070 (2)	0.5895 (16)	0.046 (6)*	
N1	0.62254 (10)	0.35391 (11)	0.53855 (9)	0.0196 (2)	
H1	0.5925 (14)	0.2927 (14)	0.5699 (12)	0.025 (4)*	
N2	0.54008 (11)	0.58808 (11)	0.63930 (8)	0.0196 (2)	
H2	0.5052 (14)	0.5435 (16)	0.6783 (11)	0.022 (4)*	
C1	0.73634 (13)	0.39280 (15)	0.60465 (11)	0.0246 (3)	
H1A	0.7760	0.4571	0.5676	0.030*	
H1B	0.7866	0.3120	0.6205	0.030*	
C2	0.72217 (14)	0.45693 (16)	0.70380 (11)	0.0267 (3)	
H2A	0.8003	0.4645	0.7507	0.032*	
H2B	0.6740	0.3967	0.7363	0.032*	
C3	0.66565 (13)	0.59536 (15)	0.69082 (10)	0.0242 (3)	
H3A	0.6738	0.6376	0.7587	0.029*	
H3B	0.7070	0.6528	0.6502	0.029*	
C4	0.48410 (14)	0.72262 (14)	0.62714 (11)	0.0244 (3)	
H4A	0.5314	0.7860	0.5964	0.029*	
H4B	0.4781	0.7584	0.6944	0.029*	
C5	0.36356 (13)	0.70700 (14)	0.55851 (10)	0.0231 (3)	
H5A	0.3146	0.6484	0.5914	0.028*	
H5B	0.3249	0.7958	0.5453	0.028*	
C6	0.31775 (13)	0.50923 (13)	0.80991 (11)	0.0203 (3)	
C7	0.18993 (17)	0.4746 (2)	0.81018 (17)	0.0289 (5)	0.822 (3)
H7A	0.1856	0.3788	0.8288	0.035*	0.822 (3)
H7B	0.1655	0.5291	0.8634	0.035*	0.822 (3)
C8	0.10391 (17)	0.49936 (19)	0.70811 (18)	0.0318 (5)	0.822 (3)
H8A	0.1308	0.4489	0.6542	0.038*	0.822 (3)
H8B	0.0263	0.4633	0.7119	0.038*	0.822 (3)
C9	0.09044 (18)	0.6455 (2)	0.67784 (15)	0.0299 (5)	0.822 (3)
H9A	0.1691	0.6840	0.6812	0.036*	0.822 (3)
H9B	0.0556	0.6942	0.7279	0.036*	0.822 (3)
C10	0.01464 (18)	0.6690 (2)	0.57143 (18)	0.0357 (5)	0.822 (3)
H10A	0.0433	0.6106	0.5227	0.043*	0.822 (3)
H10B	−0.0665	0.6411	0.5709	0.043*	0.822 (3)
C11	0.01253 (18)	0.8120 (3)	0.53458 (18)	0.0402 (6)	0.822 (3)
H11A	0.0928	0.8384	0.5306	0.048*	0.822 (3)
H11B	−0.0118	0.8714	0.5851	0.048*	0.822 (3)
C12	−0.0696 (3)	0.8339 (5)	0.4308 (2)	0.0541 (9)	0.822 (3)
H12A	−0.0671	0.9286	0.4112	0.081*	0.822 (3)
H12B	−0.1497	0.8101	0.4344	0.081*	0.822 (3)
H12C	−0.0450	0.7771	0.3799	0.081*	0.822 (3)
C7'	0.2043 (7)	0.4295 (9)	0.7967 (10)	0.0289 (5)	0.18
H7'1	0.1981	0.3868	0.8617	0.035*	0.178 (3)
H7'2	0.1995	0.3589	0.7441	0.035*	0.178 (3)
C8'	0.1079 (7)	0.5358 (9)	0.7631 (8)	0.0318 (5)	0.18
H8'1	0.0302	0.4928	0.7548	0.038*	0.178 (3)
H8'2	0.1152	0.6057	0.8165	0.038*	0.178 (3)
C9'	0.1167 (9)	0.6010 (11)	0.6632 (7)	0.0299 (5)	0.18
H9'1	0.1238	0.5297	0.6137	0.036*	0.178 (3)
H9'2	0.1889	0.6561	0.6750	0.036*	0.178 (3)
C10'	0.0116 (8)	0.6905 (12)	0.6166 (8)	0.0357 (5)	0.18
H10C	−0.0619	0.6384	0.6088	0.043*	0.178 (3)
H10D	0.0080	0.7676	0.6624	0.043*	0.178 (3)
C11'	0.0239 (9)	0.7419 (15)	0.5123 (8)	0.0402 (6)	0.18
H11C	0.0363	0.6646	0.4696	0.048*	0.178 (3)
H11D	0.0934	0.8010	0.5215	0.048*	0.178 (3)
C12'	−0.0852 (14)	0.820 (3)	0.4572 (14)	0.0541 (9)	0.18
H12D	−0.0733	0.8539	0.3918	0.081*	0.178 (3)
H12E	−0.0983	0.8964	0.4996	0.081*	0.178 (3)
H12F	−0.1536	0.7608	0.4450	0.081*	0.178 (3)

### Atomic displacement parameters (Å^2^)

**Table:**

	*U*^11^	*U*^22^	*U*^33^	*U*^12^	*U*^13^	*U*^23^
Cu1	0.02403 (13)	0.01387 (12)	0.01321 (12)	−0.00126 (8)	0.00619 (9)	−0.00200 (7)
O1	0.0350 (6)	0.0224 (5)	0.0193 (5)	0.0001 (4)	0.0071 (4)	−0.0030 (4)
O2	0.0375 (6)	0.0262 (5)	0.0170 (5)	−0.0036 (4)	0.0114 (4)	−0.0006 (4)
O1W	0.0414 (6)	0.0197 (5)	0.0214 (5)	−0.0041 (4)	0.0155 (5)	−0.0017 (4)
O2W	0.0334 (6)	0.0254 (5)	0.0243 (5)	0.0020 (4)	0.0098 (5)	−0.0037 (4)
N1	0.0278 (6)	0.0160 (5)	0.0168 (5)	−0.0017 (4)	0.0087 (5)	0.0008 (4)
N2	0.0286 (6)	0.0164 (5)	0.0154 (5)	−0.0036 (4)	0.0086 (5)	−0.0012 (4)
C1	0.0262 (7)	0.0244 (7)	0.0236 (7)	0.0000 (6)	0.0062 (6)	0.0026 (5)
C2	0.0281 (7)	0.0309 (7)	0.0195 (7)	−0.0030 (6)	0.0023 (6)	0.0020 (6)
C3	0.0296 (7)	0.0261 (7)	0.0167 (6)	−0.0071 (6)	0.0048 (5)	−0.0043 (5)
C4	0.0397 (8)	0.0157 (6)	0.0200 (7)	−0.0013 (6)	0.0114 (6)	−0.0040 (5)
C5	0.0348 (8)	0.0175 (6)	0.0201 (7)	0.0028 (5)	0.0129 (6)	0.0003 (5)
C6	0.0256 (7)	0.0181 (6)	0.0180 (6)	−0.0008 (5)	0.0067 (5)	0.0044 (5)
C7	0.0285 (9)	0.0325 (12)	0.0286 (11)	−0.0059 (9)	0.0126 (7)	0.0039 (9)
C8	0.0227 (9)	0.0334 (11)	0.0399 (13)	−0.0077 (7)	0.0084 (9)	−0.0050 (8)
C9	0.0235 (10)	0.0395 (13)	0.0263 (10)	−0.0052 (8)	0.0048 (7)	−0.0021 (8)
C10	0.0227 (8)	0.0492 (12)	0.0325 (12)	−0.0052 (8)	0.0008 (9)	−0.0009 (10)
C11	0.0225 (9)	0.0668 (17)	0.0313 (11)	0.0054 (11)	0.0064 (8)	0.0068 (11)
C12	0.0371 (14)	0.0795 (19)	0.039 (2)	0.0062 (13)	−0.0047 (11)	0.0093 (15)
C7'	0.0285 (9)	0.0325 (12)	0.0286 (11)	−0.0059 (9)	0.0126 (7)	0.0039 (9)
C8'	0.0227 (9)	0.0334 (11)	0.0399 (13)	−0.0077 (7)	0.0084 (9)	−0.0050 (8)
C9'	0.0235 (10)	0.0395 (13)	0.0263 (10)	−0.0052 (8)	0.0048 (7)	−0.0021 (8)
C10'	0.0227 (8)	0.0492 (12)	0.0325 (12)	−0.0052 (8)	0.0008 (9)	−0.0009 (10)
C11'	0.0225 (9)	0.0668 (17)	0.0313 (11)	0.0054 (11)	0.0064 (8)	0.0068 (11)
C12'	0.0371 (14)	0.0795 (19)	0.039 (2)	0.0062 (13)	−0.0047 (11)	0.0093 (15)

### Geometric parameters (Å, °)

**Table:**

Cu1—N1	2.026 (1)	C7—H7B	0.9900
Cu1—N2	2.025 (1)	C8—C9	1.507 (3)
Cu1—N2^i^	2.025 (1)	C8—H8A	0.9900
Cu1—N1^i^	2.026 (1)	C8—H8B	0.9900
Cu1—O1w	2.499 (1)	C9—C10	1.522 (3)
O1—C6	1.2584 (18)	C9—H9A	0.9900
O2—C6	1.2456 (18)	C9—H9B	0.9900
O1W—H11	0.826 (10)	C10—C11	1.504 (3)
O1W—H12	0.831 (9)	C10—H10A	0.9900
O2W—H21	0.829 (10)	C10—H10B	0.9900
O2W—H22	0.836 (10)	C11—C12	1.521 (3)
N1—C1	1.4771 (19)	C11—H11A	0.9900
N1—C5^i^	1.4818 (17)	C11—H11B	0.9900
N1—H1	0.860 (9)	C12—H12A	0.9800
N2—C3	1.4798 (18)	C12—H12B	0.9800
N2—C4	1.4827 (18)	C12—H12C	0.9800
N2—H2	0.858 (9)	C7'—C8'	1.539 (9)
C1—C2	1.521 (2)	C7'—H7'1	0.9900
C1—H1A	0.9900	C7'—H7'2	0.9900
C1—H1B	0.9900	C8'—C9'	1.516 (8)
C2—C3	1.520 (2)	C8'—H8'1	0.9900
C2—H2A	0.9900	C8'—H8'2	0.9900
C2—H2B	0.9900	C9'—C10'	1.535 (9)
C3—H3A	0.9900	C9'—H9'1	0.9900
C3—H3B	0.9900	C9'—H9'2	0.9900
C4—C5	1.511 (2)	C10'—C11'	1.531 (9)
C4—H4A	0.9900	C10'—H10C	0.9900
C4—H4B	0.9900	C10'—H10D	0.9900
C5—N1^i^	1.4818 (17)	C11'—C12'	1.538 (9)
C5—H5A	0.9900	C11'—H11C	0.9900
C5—H5B	0.9900	C11'—H11D	0.9900
C6—C7'	1.524 (8)	C12'—H12D	0.9800
C6—C7	1.539 (2)	C12'—H12E	0.9800
C7—C8	1.528 (3)	C12'—H12F	0.9800
C7—H7A	0.9900		
			
N2—Cu1—N2^i^	180.0	H7A—C7—H7B	107.7
N2—Cu1—N1^i^	85.92 (5)	C9—C8—C7	113.91 (17)
N2^i^—Cu1—N1^i^	94.08 (5)	C9—C8—H8A	108.8
N2—Cu1—N1	94.08 (5)	C7—C8—H8A	108.8
N2^i^—Cu1—N1	85.92 (5)	C9—C8—H8B	108.8
N1^i^—Cu1—N1	180.0	C7—C8—H8B	108.8
N2—Cu1—O1W	90.63 (4)	H8A—C8—H8B	107.7
N2^i^—Cu1—O1W	89.37 (4)	C8—C9—C10	113.94 (18)
N1^i^—Cu1—O1W	90.23 (4)	C8—C9—H9A	108.8
N1—Cu1—O1W	89.77 (4)	C10—C9—H9A	108.8
Cu1—O1W—H11	108.7 (16)	C8—C9—H9B	108.8
Cu1—O1W—H12	124.3 (14)	C10—C9—H9B	108.8
H11—O1W—H12	106 (2)	H9A—C9—H9B	107.7
H21—O2W—H22	103 (2)	C11—C10—C9	114.72 (19)
C1—N1—C5^i^	111.88 (11)	C11—C10—H10A	108.6
C1—N1—Cu1	117.23 (9)	C9—C10—H10A	108.6
C5^i^—N1—Cu1	106.32 (8)	C11—C10—H10B	108.6
C1—N1—H1	107.7 (12)	C9—C10—H10B	108.6
C5^i^—N1—H1	106.4 (12)	H10A—C10—H10B	107.6
Cu1—N1—H1	106.7 (12)	C10—C11—C12	113.4 (3)
C3—N2—C4	112.08 (11)	C10—C11—H11A	108.9
C3—N2—Cu1	116.85 (9)	C12—C11—H11A	108.9
C4—N2—Cu1	106.51 (8)	C10—C11—H11B	108.9
C3—N2—H2	107.5 (12)	C12—C11—H11B	108.9
C4—N2—H2	105.9 (12)	H11A—C11—H11B	107.7
Cu1—N2—H2	107.4 (12)	C11—C12—H12A	109.5
N1—C1—C2	111.97 (12)	C11—C12—H12B	109.5
N1—C1—H1A	109.2	H12A—C12—H12B	109.5
C2—C1—H1A	109.2	C11—C12—H12C	109.5
N1—C1—H1B	109.2	H12A—C12—H12C	109.5
C2—C1—H1B	109.2	H12B—C12—H12C	109.5
H1A—C1—H1B	107.9	C6—C7'—C8'	103.9 (6)
C1—C2—C3	114.22 (12)	C6—C7'—H7'1	111.0
C1—C2—H2A	108.7	C8'—C7'—H7'1	111.0
C3—C2—H2A	108.7	C6—C7'—H7'2	111.0
C1—C2—H2B	108.7	C8'—C7'—H7'2	111.0
C3—C2—H2B	108.7	H7'1—C7'—H7'2	109.0
H2A—C2—H2B	107.6	C9'—C8'—C7'	111.2 (8)
N2—C3—C2	111.77 (11)	C9'—C8'—H8'1	109.4
N2—C3—H3A	109.3	C7'—C8'—H8'1	109.4
C2—C3—H3A	109.3	C9'—C8'—H8'2	109.4
N2—C3—H3B	109.3	C7'—C8'—H8'2	109.4
C2—C3—H3B	109.3	H8'1—C8'—H8'2	108.0
H3A—C3—H3B	107.9	C8'—C9'—C10'	113.6 (8)
N2—C4—C5	107.69 (11)	C8'—C9'—H9'1	108.9
N2—C4—H4A	110.2	C10'—C9'—H9'1	108.9
C5—C4—H4A	110.2	C8'—C9'—H9'2	108.9
N2—C4—H4B	110.2	C10'—C9'—H9'2	108.9
C5—C4—H4B	110.2	H9'1—C9'—H9'2	107.7
H4A—C4—H4B	108.5	C11'—C10'—C9'	109.5 (8)
N1^i^—C5—C4	107.82 (11)	C11'—C10'—H10C	109.8
N1^i^—C5—H5A	110.1	C9'—C10'—H10C	109.8
C4—C5—H5A	110.1	C11'—C10'—H10D	109.8
N1^i^—C5—H5B	110.1	C9'—C10'—H10D	109.8
C4—C5—H5B	110.1	H10C—C10'—H10D	108.2
H5A—C5—H5B	108.5	C10'—C11'—C12'	111.7 (10)
O2—C6—O1	124.58 (14)	C10'—C11'—H11C	109.3
O2—C6—C7'	106.2 (5)	C12'—C11'—H11C	109.3
O1—C6—C7'	127.8 (5)	C10'—C11'—H11D	109.3
O2—C6—C7	120.84 (14)	C12'—C11'—H11D	109.3
O1—C6—C7	114.56 (14)	H11C—C11'—H11D	107.9
C7'—C6—C7	19.8 (4)	C11'—C12'—H12D	109.5
C8—C7—C6	113.97 (16)	C11'—C12'—H12E	109.5
C8—C7—H7A	108.8	H12D—C12'—H12E	109.5
C6—C7—H7A	108.8	C11'—C12'—H12F	109.5
C8—C7—H7B	108.8	H12D—C12'—H12F	109.5
C6—C7—H7B	108.8	H12E—C12'—H12F	109.5
			
N2—Cu1—N1—C1	−38.82 (10)	C3—N2—C4—C5	170.38 (11)
N2^i^—Cu1—N1—C1	141.18 (10)	Cu1—N2—C4—C5	41.42 (12)
O1W—Cu1—N1—C1	−129.43 (9)	N2—C4—C5—N1^i^	−56.62 (14)
N2—Cu1—N1—C5^i^	−164.78 (9)	O2—C6—C7—C8	−40.9 (2)
N2^i^—Cu1—N1—C5^i^	15.22 (9)	O1—C6—C7—C8	137.52 (16)
O1W—Cu1—N1—C5^i^	104.60 (9)	C7'—C6—C7—C8	−86.5 (15)
N1^i^—Cu1—N2—C3	−140.76 (10)	C6—C7—C8—C9	−66.1 (2)
N1—Cu1—N2—C3	39.24 (10)	C7—C8—C9—C10	174.03 (18)
O1W—Cu1—N2—C3	129.05 (9)	C8—C9—C10—C11	−172.59 (19)
N1^i^—Cu1—N2—C4	−14.63 (9)	C9—C10—C11—C12	−176.5 (2)
N1—Cu1—N2—C4	165.37 (9)	O2—C6—C7'—C8'	−105.8 (7)
O1W—Cu1—N2—C4	−104.81 (9)	O1—C6—C7'—C8'	87.6 (8)
C5^i^—N1—C1—C2	179.31 (11)	C7—C6—C7'—C8'	34.5 (10)
Cu1—N1—C1—C2	56.13 (14)	C6—C7'—C8'—C9'	60.9 (11)
N1—C1—C2—C3	−69.50 (16)	C7'—C8'—C9'—C10'	170.4 (9)
C4—N2—C3—C2	179.52 (11)	C8'—C9'—C10'—C11'	−175.3 (10)
Cu1—N2—C3—C2	−57.16 (13)	C9'—C10'—C11'—C12'	174.2 (15)
C1—C2—C3—N2	70.11 (16)		

Symmetry codes: (i) −*x*+1, −*y*+1, −*z*+1.

### Hydrogen-bond geometry (Å, °)

**Table:**

*D*—H···*A*	*D*—H	H···*A*	*D*···*A*	*D*—H···*A*
N1—H1···O1^ii^	0.86 (1)	2.30 (1)	2.983 (2)	137 (2)
N2—H2···O2	0.86 (1)	2.12 (1)	2.924 (2)	156 (2)
O1w—H11···O2	0.83 (1)	1.93 (1)	2.730 (2)	162 (2)
O1w—H12···O2w	0.83 (1)	1.95 (1)	2.777 (2)	174 (2)
O2w—H21···O1^iii^	0.83 (1)	2.02 (1)	2.833 (2)	166 (2)
O2w—H22···O1^ii^	0.84 (1)	1.91 (1)	2.743 (2)	171 (2)

Symmetry codes: (ii) −*x*+1, *y*−1/2, −*z*+3/2; (iii) *x*, −*y*+1/2, *z*−1/2.
